# Machine learning algorithm-based identification and verification of characteristic genes in acute kidney injury

**DOI:** 10.3389/fmed.2022.1016459

**Published:** 2022-10-13

**Authors:** Yinghao Li, Yiwei Du, Yanlong Zhang, Chao Chen, Jian Zhang, Xin Zhang, Min Zhang, Yong Yan

**Affiliations:** ^1^Department of Urology, Beijing Shijitan Hospital, Capital Medical University, Beijing, China; ^2^Department of Nephrology, Tangdu Hospital, Air Force Military Medical University (Fourth Military Medical University), Xi’an, China; ^3^Department of Urology, Beijing Chao-Yang Hospital, Capital Medical University, Beijing, China; ^4^Institute of Urology, Capital Medical University, Beijing, China; ^5^Department of Research Ward, Beijing Chao-Yang Hospital, Capital Medical University, Beijing, China

**Keywords:** acute kidney injury, kidney transplantation, ischemia-reperfusion injury, machine learning algorithms, RNA-seq, disease biomarker

## Abstract

**Background:**

Acute kidney injury is a common renal disease with high incidence and mortality. Early identification of high-risk acute renal injury patients following renal transplant could improve their prognosis, however, no biomarker exists for early detection.

**Methods:**

The GSE139061 dataset was used to identify hub genes in 86 DEGs between acute kidney injury and control samples using three machine learning algorithms (LASSO, random forest, and support vector machine-recursive feature elimination). We used GSEA to identify the related signal pathways of six hub genes. Finally, we validated these potential biomarkers in an *in vitro* hypoxia/reoxygenation injury cell model using RT-qPCR.

**Results:**

Six hub genes *(MDFI, EHBP1L1, FBXW4, MDM4, RALYL*, and *ESM1)* were identified as potentially predictive of an acute kidney injury. The expression of *ESM1* and *RALYL* were markedly increased in control samples, while *EHBP1L1, FBXW4, MDFI*, and *MDM4* were markedly increased in acute kidney injury samples.

**Conclusion:**

We screened six hub genes related to acute kidney injury using three machine learning algorithms and identified genes with potential diagnostic utility. The hub genes identified in this study might play a significant role in the pathophysiology and progression of AKI. As such, they might be useful for the early diagnosis of AKI and provide the possibility of improving the prognosis of AKI patients.

## Introduction

Acute kidney injury (AKI) is a common renal disease associated with high mortality and morbidity rates. At present, AKI accounts for 10–15% of complications in hospitalized patients, with an associated mortality of approximately 23.9% (1). AKI is often secondary to ischemia-reperfusion (I/R) injury following kidney transplant, major surgery, nephrotoxic drugs, and general infection (2). Simultaneously, AKI of the donor’s kidney directly impairs the recovery of renal function and organ survival following transplantation (3). AKI might develop immediately post-transplantation after initial recovery of kidney function or might occur later in recovery. In both settings, AKI can originate from unrecognized severe clinical conditions that require prompt intervention to prevent graft loss. Such prompt responses can improve the prognosis of patients with AKI, which relies on early diagnosis (4). At present, the main diagnostic criteria for AKI include the “risk, injury, failure” (RIFLE) standard developed by the acute dialysis quality initiative group (5), the improved RIFLE standard from the AKI network (6), and the kidney disease: Improving Global Outcomes (KDIGO) standard (7). Although all three are widely used clinically, there remain controversies in predicting delayed graft function or post-transplant outcomes.

Research studies have gradually deepened our understanding of the pathophysiology and molecular biological mechanisms underlying AKI (8, 9). Multiple mechanisms have been proposed to be involved in AKI pathophysiology, such as ischemia-reperfusion injury, inflammation, autophagy, and oxidative stress, but their relationships have not been thoroughly investigated (10). The pathogenesis of AKI involves injury to renal tubular cells, glomerular cells, and renal interstitial cells and can be classified as prerenal, renal, or postrenal. The mechanisms of injury include apoptosis, proptosis, autophagy, oxidative stress, etc. However, these cellular events occur following the initiation of AKI; once these molecular mechanisms are initiated, cells are beyond rescue. As such, it is critical to identify the molecular features involved in initiating AKI to facilitate the development of effective therapeutic strategies for halting AKI.

Many studies have attempted to identifies early AKI diagnostic biomarkers. Parikh CR et al. identified kidney injury molecule-1 (KIM-1) as a biomarker of acute kidney injury (11); compared with creatinine, the urine concentration of KIM-1 rises within 24h of AKI onset. It has been demonstrated that *TIMP-2* and *IGFBP7* are useful in predicting acute kidney injury following cardiac surgery (12), and compared with other biomarkers, *TIMP-2* and *IGFBP7* might be useful to predict the onset of severe AKI. In addition, other biomarkers have been tested in preclinical settings. Compared with patients without AKI after kidney transplantation, there is an increase of secretory leucocyte peptidase inhibitor (*SLPI*) in AKI patient plasma and urine; *SLPI* has a unique significance in transplantation-related AKI (13), and in the perfusion solution. It may help to identify graft quality after kidney transplantation (14). In the mouse model of ischemic AKI, ischemia can upregulate the expression of macrophage migration inhibitor 2, thereby promoting the release of *SLPI* and imparting renal protection. In addition, *SLPI* mRNA is shown to be significantly up-regulated in renal biopsies of AKI patients during the early stage after renal transplantation, compared with patients not affected by post-transplant AKI (15). However, the availability of sensitive and specific biomarkers related to the etiology of AKI is limited. With the development of high-throughput sequencing and bioinformatics, we can analyze the molecular features of AKI and quickly select relevant disease markers from the genome. Compared with traditional research approaches, we can further investigate the molecular characteristics of AKI by applying these techniques (16, 17). The detection of AKI-related biomarkers might be more able to reflect the pathophysiological processes underlying AKI and is expected to become a means of diagnosis and evaluation of AKI in the future.

In this study, mRNA microarray data was used to explore the molecular features of AKI. We further identified the AKI signature using machine learning. Finally, we validated these potential biomarkers *via* an *in vitro* hypoxia/reoxygenation injury model.

## Materials and methods

### Collection and processing of acute kidney injury datasets

A study flowchart was shown in Supplementary Figure 1. The mRNA sequencing and its clinical data associated with AKI samples and control samples (Supplementary Table 1) were obtained from the Gene Expression Omnibus (GEO) database (GSE139061)^1^ (18), and there was no significant difference in age (*p* = 0.27) and gender (*p* = 0.33) between the AKI samples and control samples in this study. It included high throughput RNA sequencing data from 39 AKI and 9 control samples; the platform of this data set was GPL20301 [Illumina HiSeq 4000 (Homo sapiens)]. The count values were normalized by log2(n+1) (Supplementary Figure 2).

### Differentially expressed genes and gene enrichment analysis

The limma package (version 3.52.0) was used to identify differentially expressed genes (DEGs) between AKI and control samples (19); the Wilcox test was used to calculate the *p*-values with significant DEGs defined by the absolute values of log fold change ≥ 1 (| logFC | ≥ 1, it means the log fold change of genes from AKI samples and control samples) and adjusted *p*-values < 0.05. Then the associated pathway and process enrichment analysis of DEGs were carried out using Metascape^2^ (20, 21).

### Hub genes selection based on machine learning algorithms

Three machine learning algorithms were used to select the gene signatures associated with AKI, including SVM Recursive Feature Elimination (SVM-RFE) (22), LASSO Model (23), and Random Forest Model (24).

SVM-RFE algorithm was applied using “rfe” in the R package “caret” (version 6.0-92); SVM-RFE is widely used to rank features and select gene signatures. To validate the SVM-RFE model, we used tenfold cross-validation to select the AKI-related gene signatures. The LASSO model is a dimensionality reduction method for evaluating high-dimensional data. A LASSO model was fitted using the “cv.glmnet” function in the R package “glmnet” (version 4.1-4). R package “randomForest” (version 4.7-1.1) was used to build the random forest model, which is a supervised nonparametric classification method. Finally, the AKI-related hub genes were obtained by the intersecting genes of the above three machine learning models. Finally, the receiver operating characteristic (ROC) curves and the area under the curve (AUC) were used to evaluate the diagnostic efficacy; the 95% confidence intervals (1,000 iterations) of the AUC were estimated by the bootstrap algorithm (25).

### Construction of the nomogram model

We created a nomogram model to predict AKI using the R package “rms” (version: 6.3-0). The expression of each gene has a corresponding point. The “Total Points” reflected the sum of all the above elements.

### Gene set enrichment analysis

Using the function “gseKEGG” of the R package “ClusterProfiler” (version 4.4.4), GSEA was performed to investigate the potential functions of the hub genes with the following parameters (nPerm = 10,000, minGSSize = 10, maxGSSize = 200, pvalueCutoff = 0.05, pAdjustMethod = “none”) (26).

### Cell culture and treatment

The human renal proximal tubular cell line HK-2 was obtained from the China Center for Type Culture Collection (GDC0152, Wuhan, China) and cultivated in DMEM/F-12 supplemented with 10% FBS, 100 U/ml penicillin, and 100 μg/ml streptomycin in a humidified atmosphere of 5% CO_2_ at 37°C. The cells were plated in six-well plates and were treated at 80–90% confluence. The cells in the hypoxia and reoxygenation (H/R) groups were cultured with glucose-free and serum-free medium for 6h under hypoxic conditions (1% O_2_, 94% N_2_, and 5% CO_2_) with following regular culture treatment with oxygen for 3, 6, and 12h in a general incubator (5% CO_2_ and 95% air), respectively (27, 28). Subsequently, cells were collected at the indicated time points mentioned above for further verification.

### Real-time quantitative PCR analysis

Total RNA extracted from cells following treatment using an M5 Universal RNA Mini Kit (MF-033-01, Mei5 Biotechnology, Beijing, China); 500 ng of RNA was reverse transcribed for cDNA synthesis. The primer sequences used in this study are summarized in Supplementary Table 2. Real-time quantitative PCR (RT-qPCR) was used to assess the relative expression level of genes compared to actin. Relative gene expression expression was calculated using 2^–ΔΔCt^.

### Statistical analysis

Unpaired Student’s *t*-test was used to compare two groups with normally distributed variables, while Mann–Whitney U-test was used to compare two groups with non-normally distributed variables. For comparisons of three groups, one-way analysis of variance and Kruskal–Wallis tests of variance were used as parametric and nonparametric methods, respectively. The Chi-square or Fisher’s exact tests were used to analyze variables in the contingency table. Statistical significance was defined as a two-tailed *p* < 0.05. R software was used to conduct all statistical analyses (version 4.1.0; RStudio, Boston, MA, USA).

## Results

### Identification and gene enrichment analyses of differentially expressed genes

**FIGURE 1:**
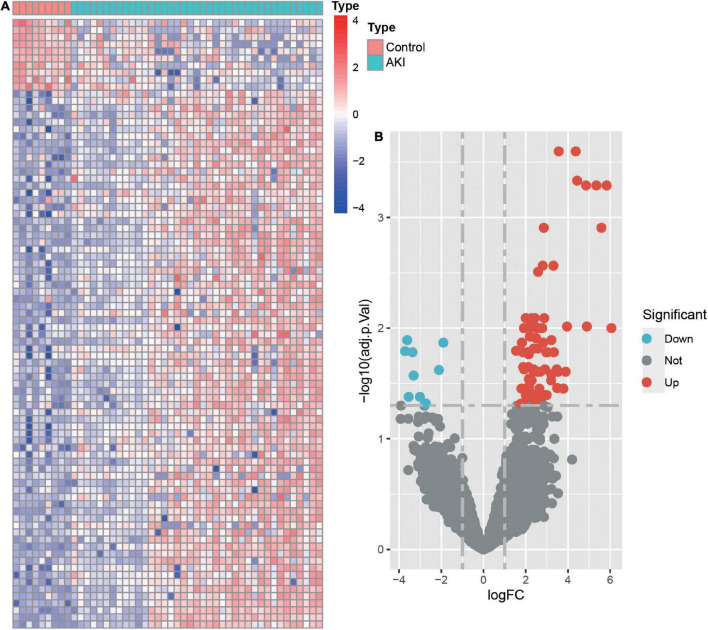
The heatmap and volcano plot of differentially expressed genes (DEGs). **(A)** Heatmap of DEGs. The vertical axis represents the samples, and the horizontal axis represents differentially expressed genes. Red indicates upregulated genes, and blue represents downregulated genes. **(B)** Volcano plot of DEGs. The *x*-axis represents the log FC, and the *y*-axis represents the -log10 (adjusted *p*-value). The blue dots represent downregulated genes, and the red dots represent upregulated genes.

**FIGURE 2:**
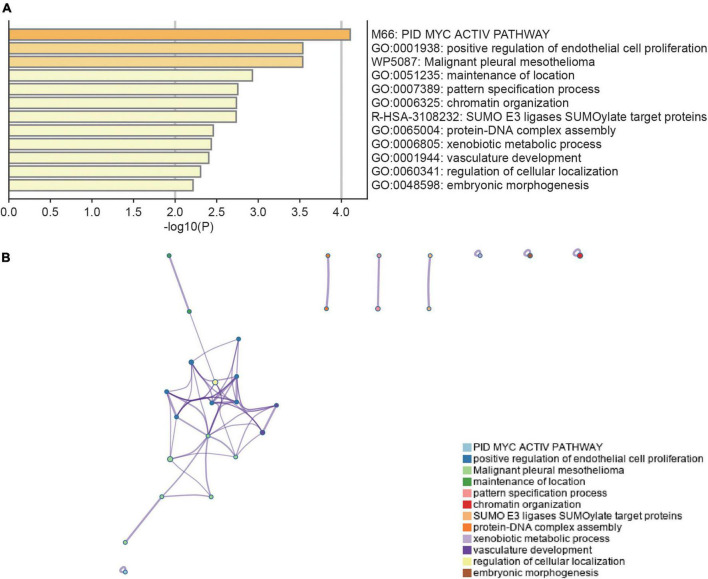
Functional enrichment analysis of DEGs. **(A)** Bar chart of functional enrichment terms colored by *p*-values (the darker the color, the smaller the *p*-value). **(B)** The functional enrichment ontology clustering graph represents each term as a circle, and each cluster has a unique color, which means that circles of the same color are associated with the same cluster. The edges connect terms that have a similarity score of >0.3 which influences the density of the edge line. Metascape (http://metascape.org) was utilized for visualization.

Differential expression analysis was conducted for 16,092 genes. By using our identification criteria, we identified 86 DEGs between the 39 AKI and 9 control samples. Among them, there were 10 down-regulated genes and 76 up-regulated genes (Supplementary Table 1). A heatmap (Figure 1A) and a volcano plot (Figure 1B) were used to visualize down-regulated genes and up-regulated genes in this study. Metascape was used to conduct enrichment analysis of these DEGs to gain a deeper understanding of their biological functions and characteristics [(Figures 2A,B) see text footnote 2]. The results of this were summarized in Supplementary Table 3 (21, 29). We found 40 terms with *p* < 0.05, including the PID MYC ACTIV PATHWAY (M66), positive regulation of endothelial cell proliferation (GO:0001938), maintenance of location (GO:0051235), and SUMO E3 ligases SUMOylate target proteins (R-HSA-3108232). It appeared that these DEGs are closely related to the composition and function of cells. The enrichment ontology cluster graph and its relationship were shown in Figure 2B.

### Hub genes selection based on support vector machine recursive feature elimination, LASSO, and random forest

**FIGURE 3:**
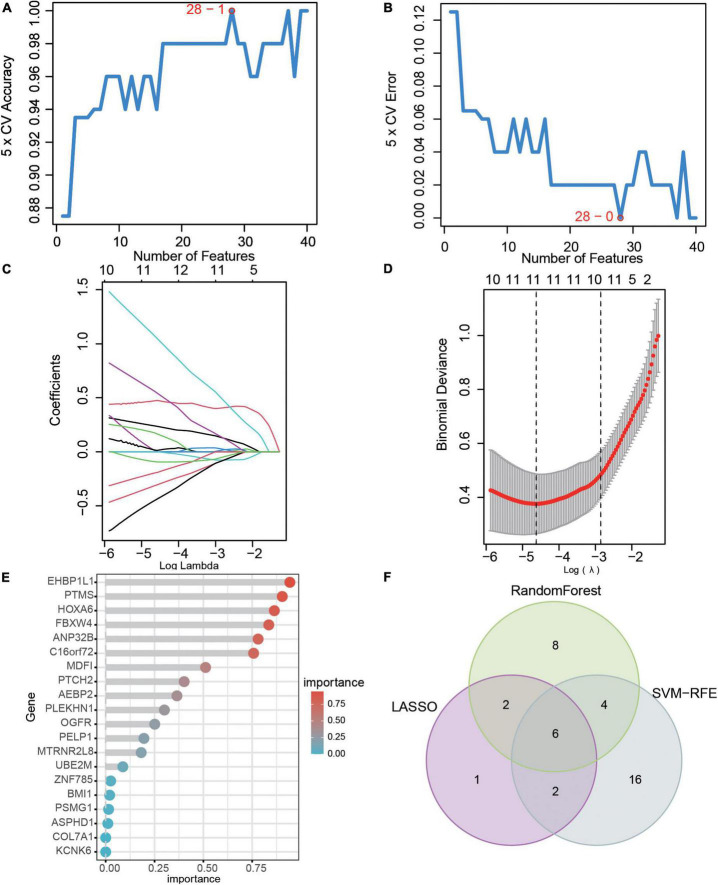
Hub genes selection based on machine learning algorithms. **(A)** The accuracy and **(B)** the error of the feature selection for the SVM-RFE algorithm. **(C)** 10-flod cross-validation for tuning parameter selection in the LASSO model. Each curve corresponds to a single gene. **(D)** LASSO coefficient profiles of DEGs. The solid vertical lines represent the partial likelihood of deviance SE. The dotted vertical line is drawn at the optimal Lambda. **(E)** The rank of genes by their relative importance in the random forest algorithm. **(F)** Venn diagram showing the characteristic genes shared by LASSO, random forest, and SVM-RFE algorithms.

Three machine learning algorithms were used to select hub genes in 86 DEGs between AKI and control samples. In the SVM-RFE model, we found that when the number of features is 28, the maximum accuracy of the classifier was 1 (Figure 3A), and the minimum error of the classifier was 0 (Figure 3B), which include *AGTR1, RALYL, GPC2, UPF2, EHBP1L1, EGFL7, ESM1, TRIM65, MDM4, FBXW4, PODXL, ADORA2A, AEBP2, ARL6IP6, KCNK6, PSMG1, TMEM255B, HOXC8, ANP32B, TEAD4, PTCH2, EGF, BTBD19, MDFI, TEN1-CDK3, CHTF8, ATAD2*, and *TNK2*. In the LASSO model, after ten-fold cross-validation, there were 11 characteristic genes (Figures 3C,D), including *MDFI, PTMS, EHBP1L1, FBXW4, MDM4, AGTR1, UBAP1L, NAP1L1, PODXL, RALYL*, and *ESM1*. In the random forest model, 20 characteristic genes were determined with importance > 0.25 (Figure 3E), including *EHBP1L1, PTMS, HOXA6, FBXW4, ANP32B, C16orf72, MDM4, UBAP1L, MDFI, PTCH2, ANK3, RALYL, AEBP2, RRP9, PLEKHN1, GSTA1, UPF2, CA11, ESM1*, and *OGFR*. Finally, we selected the intersection of the results of the above three algorithms which delivered the hub genes *MDFI, EHBP1L1, FBXW4, MDM4, RALYL*, and *ESM1*. We visualized the hub genes with a veen diagram (Figure 3F).

### Modeling of an acute kidney injury diagnostic nomogram

We built an AKI diagnostic nomogram (Supplementary Figure 3A) for the hub genes (*MDFI, EHBP1L1, FBXW4, MDM4, RALYL*, and *ESM1*). Then, the diagnostic efficiency of the nomogram was evaluated by using ROC and AUC, we found that the AUC = 1 of the nomogram (Supplementary Figure 3B). The coefficients of this nomogram were in the Supplementary Table 4, and the total points could be calculated as follows:


T⁢o⁢t⁢a⁢l⁢P⁢o⁢i⁢n⁢t⁢s=∑n*c⁢o⁢e⁢f⁢f⁢c⁢i⁢e⁢n⁢t⁢(n⁢m⁢e⁢a⁢n⁢s⁢h⁢u⁢b⁢g⁢e⁢n⁢e⁢s)

### The diagnostic efficacy of hub genes in predicting acute kidney injury

**FIGURE 4:**
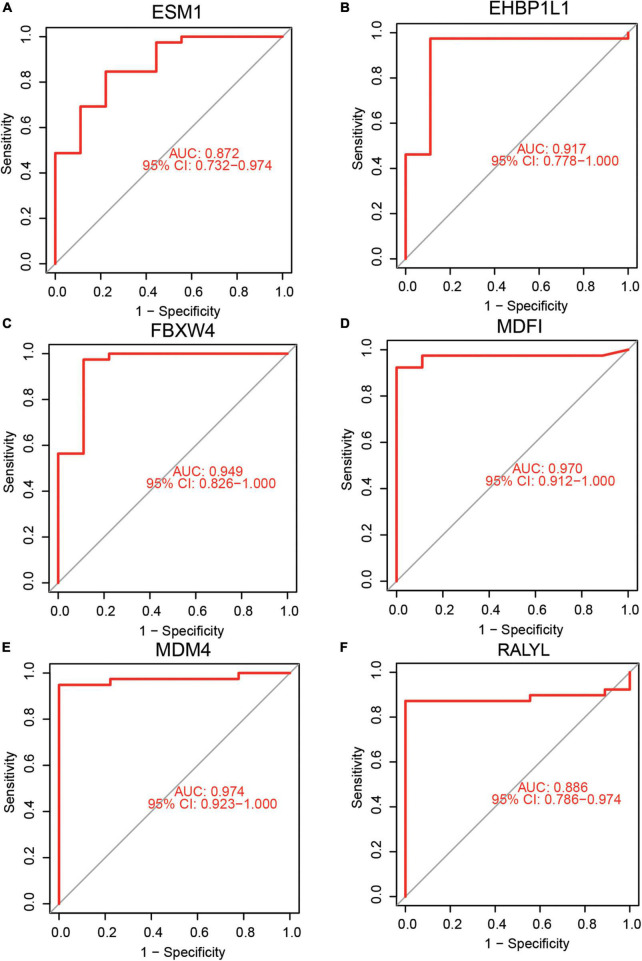
Diagnostic efficacy of hub genes in the prediction of AKI. **(A)** *ESM1* AUC = 0.872 (95%CI: 0.732–0.974), **(B)** *EHBP1L1* AUC = 0.917 (95%CI: 0.778–1.000), **(C)** *FBXW4* AUC = 0.949 (95%CI: 0.826–1.000), **(D)** *MDFI* AUC = 0.970 (95%CI: 0.912–1.000), **(E)** *MDM4* AUC = 0.974 (95%CI: 0.923–1.000), **(F)** *RALYL* AUC = 0.886 (95%CI: 0.786–0.974).

Receiver operating characteristic and AUC were used to evaluate the diagnostic efficacy of the six identified hub genes in predicting AKI. These showed a potential efficacy in predicting AKI: the AUC were 0.872 (95% CI 0.732–0.974) for *ESM1* (Figure 4A), 0.917 (95%CI: 0.778–1.000) for *EHBP1L1*(Figure 4B), 0.949 (95%CI: 0.826–1.000) for *FBXW4* (Figure 4C), 0.970 (95%CI: 0.912–1.000) for *MDFI* (Figure 4D), 0.974 (95%CI: 0.923–1.000) for *MDM4* (Figure 4E), and 0.886 (95%CI: 0.786–0.974) for *RALYL* (Figure 4F). Then, cutoff values for hub genes were calculated by ROC analysis and were shown in Supplementary Table 4. Some calculations were made according to the cutoff values including calculation of the sensitivity values, specificity values, positive predictive values, and negative predictive values, *MDFI* (cutoff value = 5.04, sensitivity value = 0.93, specificity value = 1.00, positive predictive value = 1.000, negative predictive value = 0.75), *EHBP1L1* (cutoff value = 9.32, sensitivity value = 0.97, specificity value = 0.89, positive predictive value = 0.97, negative predictive value = 0.89), *FBXW4* (cutoff value = 8.91, sensitivity value = 0.97, specificity value = 0.89, positive predictive value = 0.97, negative predictive value = 0.89), *MDM4* (cutoff value = 10.14, sensitivity value = 0.95, specificity value = 1.00, positive predictive value = 1.000, negative predictive value = 0.82), *RALYL* (cutoff value = 6.45, sensitivity value = 0.87, specificity value = 1.00, positive predictive value = 1.000, negative predictive value = 0.65), and *ESM1* (cutoff value = 4.97, sensitivity value = 0.85, specificity value = 0.78, positive predictive value = 0.94, negative predictive value = 0.54).

### Signaling pathways related to the hub genes

**FIGURE 5:**
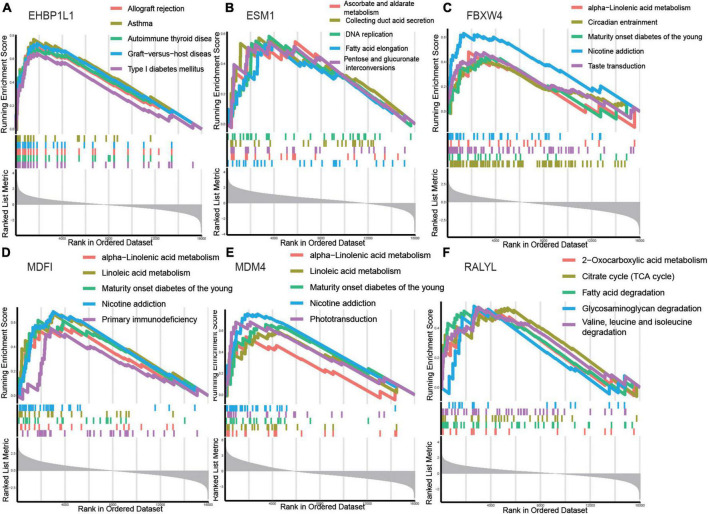
Gene set enrichment analysis (GSEA) identifies signaling pathways involved in the hub genes. **(A–F)** The main signaling pathways that are significantly enriched in high expressions of hub genes. **(A)** *EHBP1L1*, **(B)** *ESM1*, **(C)** *FBXW4*, **(D)** *MDFI*, **(E)** *MDM4*, **(F)** *RALYL*.

Gene set enrichment analysis was used to identify the relevant signaling pathways of the six hub genes. *EHBP1L1* (Figure 5A) was positively linked to allograft rejection, asthma, autoimmune thyroid disease, graft–versus–host disease, and type I diabetes mellitus. *ESM1* (Figure 5B) was positively linked to ascorbate and aldarate metabolism, collecting duct acid secretion, DNA replication, fatty acid elongation, and pentose and glucuronate interconversions. *FBXW4* (Figure 5C) was positively linked to alpha–linolenic acid metabolism, circadian entrainment, maturity onset diabetes of the young, nicotine addiction, and taste transduction. *MDFI* (Figure 5D) was positively linked to alpha–linolenic acid metabolism, linoleic acid metabolism, maturity onset diabetes of the young, nicotine addiction, and primary immunodeficiency. *MDM4* (Figure 5E) was positively linked to alpha–linolenic acid metabolism, linoleic acid metabolism, maturity onset diabetes of the young, nicotine addiction, and phototransduction. *RALYL* (Figure 5F) was positively linked to 2–oxocarboxylic acid metabolism, citrate cycle (TCA cycle), fatty acid degradation, glycosaminoglycan degradation, and valine, leucine and isoleucine degradation.

The results showed that the hub genes were related to metabolism (ascorbate and aldarate metabolism, alpha–linolenic acid metabolism, linoleic acid metabolism, 2–oxocarboxylic acid metabolism, citrate cycle (TCA cycle), and fatty acid degradation), which might play an important role in the pathogenesis and progression of AKI.

### Hub genes expression and RT-qPCR validation

**FIGURE 6:**
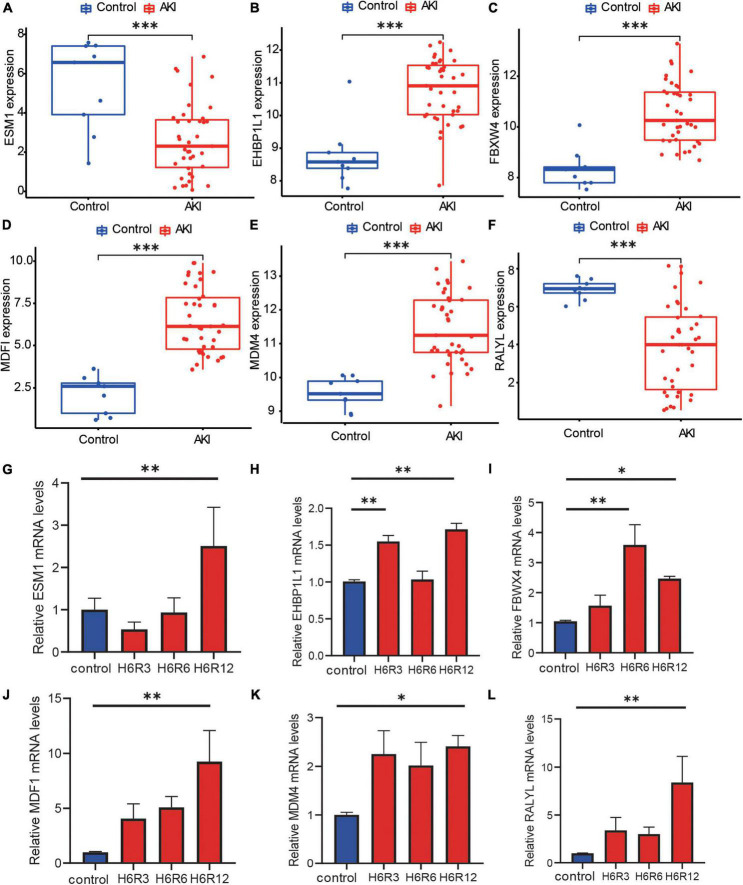
The expression level of hub genes and RT-qPCR validation. **(A–F)** Box plots showed the mRNA expression level of hub genes between AKI samples and control samples, and the *p*-value was marked below the horizontal line in the figure. **(A)** *ESM1* (*p* = 0.001), **(B)** *EHBP1L1* (*p* < 0.001), **(C)** *FBXW4* (*p* < 0.001), **(D)** *MDFI* (*p* < 0.001), **(E)** *MDM4* (*p* < 0.001), **(F)** *RALYL* (*p* < 0.001). **(G–L)** Box plots for Real-time PCR validation among hub genes, Kruskal–Wallis test, *p < 0.05, ^**^p < 0.01, and ^***^p < 0.001.

Six hub genes were analyzed for their expression levels (Figures 6A–F). Expression of *ESM1* (Figure 6A, Mann–Whitney U-test, *p* = 0.001) and *RALYL* (Figure 6F, Mann–Whitney U-test, *p* < 0.001) was markedly increased in control samples. The expressions of *EHBP1L1* (Figure 6B, Mann–Whitney U-test, *p* < 0.001), *FBXW4* (Figure 6C, Mann–Whitney U-test, *p* < 0.001), *MDFI* (Figure 6D, Mann–Whitney U-test, *p* < 0.001), and *MDM4* (Figure 6E, Mann–Whitney U-test, *p* < 0.001), were markedly increased in AKI samples. The difference in these hub genes may be involved in AKI disease onset and progression.

We then used RT-qPCR to verify these findings. After reoxygenation, *ESM1* expression trended lower than in the control group (Figure 6G) but the difference was not significant. However, after 12h of reoxygenation, *ESM1* expression was increased. After reoxygenation, the expression of *EHBP1L1* (Figure 6H), *FBWX4* (Figure 6I), *MDFI* (Figure 6J), and *MDM4* (Figure 6K) was significantly higher (Kruskal–Wallis test, *p < 0.05, ^**^p < 0.01). These results support the reliability of our findings and underpin their future utility. After reoxygenation, *RALYL* expression was higher than in the control group (Figure 6L), which is contrary to our prediction. Further investigation is required to explain this finding.

## Discussion

AKI was a clinical syndrome caused by acute decline or loss of renal filtration function stemming from multiple causes (10) and was associated with high morbidity and mortality (30). AKI was also a serious complication of other severe conditions (31). Most AKI develops gradually and manifests with a variety of pathophysiological symptoms and signs across a spectrum spanning mild decline of renal filtration function to the need for renal replacement therapy. Ischemia and hypoxia were the main triggers of AKI (32). As AKI progresses, ischemia and hypoxia further damage the morphology and functional anabolism of renal tubular epithelial cells and renal fibroblasts, resulting in delayed recovery and the loss of renal tubule reabsorption and secretion function. In addition, renal microvascular endothelial growth factor levels decrease, further reducing the number of capillaries, aggravating hypoxia, and eventually resulting in chronic kidney disease (CKD) (4). Renal transplantation was the most effective treatment of end-stage renal disease (*ESRD*), but ischemia-reperfusion injury may occur during the process of kidney organ donation and transplantation, resulting in AKI (33). As such, the prevention of postoperative AKI relied on being able to reliably quantify its risk following renal transplantation.

In this study, we used three machine learning algorithms to select hub genes in 86 DEGs between AKI and control samples by using the GSE139061 dataset. Then, we selected common genes from the three algorithms which were the hub genes *MDFI, EHBP1L1, FBXW4, MDM4, RALYL*, and *ESM1*. The AUC of these genes ranged from 0.872 to 0.974, indicating a high level of predictive power for AKI, and the AUC of the nomogram was 1, we also used the bootstrap algorithm to estimate the 95% AUC confidence intervals. We quantified the traditional AKI diagnostic markers such as KIM-1, Cystatin C, NGAL, IL-18 and their associated protein-coding genes (*HAVCR1, LCN2, IL18, CYCS, and CST3*) and assessed the diagnostic performance of these genes using ROC and AUC. The AUCs were 0.638 (95% CI 0.453–0.806) for *HAVCR1* (Supplementary Figure 4A), 0.613 (95%CI: 0.430–0.781) for *LCN2* (Supplementary Figure 4B), 0.749 (95%CI: 0.550–0.917) for *IL18* (Supplementary Figure 4C), 0.554 (95%CI: 0.276–0.798) for *CYCS* (Supplementary Figure 4D), and 0.581 (95%CI: 0.305–0.832) for *CST3* (Supplementary Figure 4E). We found that while these genes may contribute to the diagnosis of AKI, their diagnostic efficacy was slightly lower than the hub genes identified in this study. Thus, we believed that the diagnostic model developed in this study showed a marked potential for clinical application in the early identification of AKI. We used GSEA to identify the relevant signaling pathways of the six hub genes. As a result, we found that the hub genes were associated with metabolism, including ascorbate metabolism, alpha–linolenic acid metabolism, and fatty acid degradation, which may contribute to the pathogenesis and progression of AKI. This showed that the metabolic state of AKI renal cells was different from controls; this may be related to the ischemic and hypoxic state of renal cells in AKI (32).

Previous studies had linked some of these hub genes with kidney disease. *EHBP1L1* regulates the CpG methylation of renal cells and was associated with the prognosis of renal clear cell carcinomas (34). The exact mechanisms of *EHBP1L1* were not elucidated and further research was needed to verify our findings. *MDM4* mRNA expression was increased in clear cell renal cell carcinomas (ccRCC); as such, it was a prognostic marker and its silencing inhibits the migration and invasiveness of RCC cells (35–38). Similarly, *RALYL* was related to the prognosis of RCC (39); its expression was significantly reduced in RCC, and this was associated with poor prognosis. However, there our findings into *RALYL* expression were conflicted between RNA-sequencing and qPCR. It had been indicated that around 15% of genes showed inconsistent results between RNA sequencing and RT-qPCR data (40). As such, further investigations were required to explain these findings. The expression of *ESM1*, also known as Endocan, was associated with adverse clinical outcomes in renal insufficiency, including AKI. As a marker of endothelial dysfunction, it was important in glomerular/vascular disease, which may lead to AKI (41). Some studies had shown the value of *ESM1* in diagnosing acute rejection after renal transplantation. *ESM1* was observed in circulating endothelial cells in the peripheral blood and in renal allografts after renal transplantation; the expression of *ESM1* mRNA and protein was found to be significantly higher in patients with acute rejection (42). Although there was no evidence that the *MDFI* and *FBXW4* genes are associated with kidney disease, *MDFI* can regulate the WNT signaling pathway, which is essential for the development of the mammalian kidney (43), and it was a cysteine rich glycoprotein in the extracellular matrix and played a key role in embryonic development and adult tissue homeostasis (44), Wnt/ β- Catenin signal was involved in the regulation of glomerulosclerosis and podocyte dysfunction, it was related to renal fibrosis. WNT was relatively silent in the normal adult kidney but played an important role in renal protection or pathogenesis when it comes to AKI (45). The role of *FBXW4* in AKI needs further research to confirm.

Although we screened the hub genes related to AKI using bioinformatics approaches and verified their diagnostic value, some limitations should be noted. Firstly, our samples were of a one-off cross-sectional nature. Because we did not perform longitudinal analyses, we were not able to test the predictive value of these hub genes on patient prognosis. Secondly, this study lacked our own clinical samples. Therefore, we need to conduct research with a larger clinical sample size and additional experiments to further verify our findings.

## Conclusion

In this study, we identified six hub genes related to AKI based on three machine learning algorithms; their AUCs showed potential diagnostic utility in predicting AKI. The hub genes identified in this study might play a significant role in the pathophysiology of AKI. They might be exploited for the early diagnosis of AKI and help improve AKI prognosis.

## Data availability statement

Publicly available datasets were analyzed in this study. This data can be found here: https://www.ncbi.nlm.nih.gov/geo/.

## Ethics statement

Ethical review and approval was not required for the study on human participants in accordance with the local legislation and institutional requirements. Written informed consent for participation was not required for this study in accordance with the national legislation and the institutional requirements.

## Author contributions

YL, YD, and YZ designed and conducted the study and drafted the manuscript. YY, XZ, and MZ revised the manuscript. YL, YD, YZ, CC, and JZ performed the data analysis. All authors have read and approved the final manuscript.

## Footnotes

1
https://www.ncbi.nlm.nih.gov/geo/

2
http://metascape.org

## Abbreviations

AKI: acute kidney injury
AUC: area under the curve
CKD: chronic kidney disease
DEGs: differentially expressed genes
ESRD: end-stage renal disease
GEO: gene expression omnibus
PCR: polymerase chain reaction
RT-qPCR: real time quantitative PCR
RCC: renal cell carcinoma
ROC: receiver operating characteristic
SVM-RFE: support vector machine recursive feature elimination.

## References

[1] Lameire NH Bagga A Cruz D De Maeseneer J Endre Z Kellum JA Acute kidney injury: an increasing global concern. *Lancet.* (2013) 382:170–9. 23727171 10.1016/S0140-6736(13)60647-9

[2] Billings FTT. Acute kidney injury following cardiac surgery: a clinical model. *Nephron.* (2019) 143:202–6. 31269500 10.1159/000501559 PMC6821568

[3] Cooper JE Wiseman AC. Acute kidney injury in kidney transplantation. *Curr Opin Nephrol Hypertens.* (2013) 22:698–703. 24076557 10.1097/MNH.0b013e328365b388

[4] Zarbock A Kellum JA Schmidt C Van Aken H Wempe C Pavenstädt H Effect of early vs delayed initiation of renal replacement therapy on mortality in critically Ill patients with acute kidney injury. *JAMA.* (2016) 315:2190. 10.1001/jama.2016.5828 27209269

[5] Bellomo R Ronco C Kellum JA Mehta RL Palevsky P. Acute renal failure – definition, outcome measures, animal models, fluid therapy and information technology needs: the second international consensus conference of the acute dialysis quality initiative (ADQI) group. *Crit Care.* (2004) 8:R204–12. 10.1186/cc2872 15312219 PMC522841

[6] Mehta RL Kellum JA Shah SV Molitoris BA Ronco C Warnock DG Acute kidney injury network: report of an initiative to improve outcomes in acute kidney injury. *Crit Care.* (2007) 11:R31. 10.1186/cc5713 PMC2206446 17331245

[7] Yang S Chiou TT Shiao C Lin HY Chan M Wu C Nomenclature and diagnostic criteria for acute kidney injury – 2020 consensus of the Taiwan AKI-task force. *J Formos Med Assoc.* (2022) 121:749–65. 10.1016/j.jfma.2021.08.005 34446340

[8] Mami I Tavernier Q Bouvier N Aboukamis R Desbuissons G Rabant M A novel extrinsic pathway for the unfolded protein response in the kidney. *J Am Soc Nephrol.* (2016) 27:2670–83. 10.1681/ASN.2015060703 26823555 PMC5004651

[9] Rabb H Griffin MD Mckay DB Swaminathan S Pickkers P Rosner MH Inflammation in AKI: current understanding, key questions, and knowledge gaps. *J Am Soc Nephrol.* (2016) 27:371–9. 10.1681/ASN.2015030261 26561643 PMC4731128

[10] Bonventre JV Yang L. Cellular pathophysiology of ischemic acute kidney injury. *J Clin Investig.* (2011) 121:4210–21. 22045571 10.1172/JCI45161 PMC3204829

[11] Parikh CR Thiessen-Philbrook H Garg AX Kadiyala D Shlipak MG Koyner JL Performance of kidney injury molecule-1 and liver fatty acid-binding protein and combined biomarkers of AKI after cardiac surgery. *Clin J Am Soc Nephrol.* (2013) 8:1079–88. 23599408 10.2215/CJN.10971012 PMC3700701

[12] Meersch M Schmidt C Hoffmeier A Van Aken H Wempe C Gerss J Prevention of cardiac surgery-associated AKI by implementing the KDIGO guidelines in high risk patients identified by biomarkers: the PrevAKI randomized controlled trial. *Intens Care Med.* (2017) 43:1551–61. 10.1007/s00134-016-4670-3 PMC5633630 28110412

[13] Wilflingseder J Sunzenauer J Toronyi E Heinzel A Kainz A Mayer B Molecular pathogenesis of post-transplant acute kidney injury: assessment of whole-genome mRNA and miRNA profiles. *PLoS One.* (2014) 9:e104164. 10.1371/journal.pone.0104164 25093671 PMC4122455

[14] Ambrosi NG Caro FY Osella F Alvarez LD Sanchez F Toniolo F SLPI in the perfusion solution helps to identify graft quality in kidney transplants. *Biomark Med.* (2019) 13:895–906. 10.2217/bmm-2018-0428 31379196

[15] Averdunk L Fitzner C Levkovich T Leaf DE Sobotta M Vieten J Secretory leukocyte protease inhibitor (SLPI)-a novel predictive biomarker of acute kidney injury after cardiac surgery: a prospective observational study. *J Clin Med.* (2019) 8:1931. 10.3390/jcm8111931 31717603 PMC6912354

[16] Cai Q Sun Z Bao M Guo S Wu H Yu X. POS-063 disulfiram ameliorates ischemia/reperfusion-induced acute kidney injury by suppressing the caspase-11-GSDMD pathway. *Kidney Int Rep.* (2022) 7:S26–7. 10.1080/0886022X.2022.2098764 35837696 PMC9291718

[17] Zhai X Lou H Hu J. Five-gene signature predicts acute kidney injury in early kidney transplant patients. *Aging.* (2022) 14:2628–44. 10.18632/aging.203962 35320116 PMC9004575

[18] Janosevic D Myslinski J Mccarthy TW Zollman A Syed F Xuei X The orchestrated cellular and molecular responses of the kidney to endotoxin define a precise sepsis timeline. *Elife.* (2021) 10:e62270. 10.7554/eLife.62270 33448928 PMC7810465

[19] Ritchie ME Phipson B Wu D Hu Y Law CW Shi W limma powers differential expression analyses for RNA-sequencing and microarray studies. *Nucleic Acids Res.* (2015) 43:e47. 10.1093/nar/gkv007 25605792 PMC4402510

[20] Subramanian A Tamayo P Mootha VK Mukherjee S Ebert BL Gillette MA Gene set enrichment analysis: a knowledge-based approach for interpreting genome-wide expression profiles. *Proc Natl Acad Sci U.S.A.* (2005) 102:15545–50. 16199517 10.1073/pnas.0506580102 PMC1239896

[21] Zhou Y Zhou B Pache L Chang M Khodabakhshi AH Tanaseichuk O Metascape provides a biologist-oriented resource for the analysis of systems-level datasets. *Nat Commun.* (2019) 10:1523. 10.1038/s41467-019-09234-6 30944313 PMC6447622

[22] Krishnapuram B Hartemink AJ Carin L Figueiredo MAT. A Bayesian approach to joint feature selection and classifier design. *IEEE Trans Pattern Anal Mach Intell.* (2004) 26:1105–11. 15742887 10.1109/TPAMI.2004.55

[23] Friedman J Hastie T Tibshirani R. Regularization paths for generalized linear models via coordinate descent. *J Stat Softw.* (2010) 33:1–22. 20808728 PMC2929880

[24] Patil KR Nielsen J. Uncovering transcriptional regulation of metabolism by using metabolic network topology. *Proc Natl Acad Sci U.S.A.* (2005) 102:2685–9. 15710883 10.1073/pnas.0406811102 PMC549453

[25] Carpenter J Bithell J. Bootstrap confidence intervals: when, which, what? A practical guide for medical statisticians. *Stat Med.* (2000) 19:1141–64. 10.1002/(sici)1097-0258(20000515)19:9<1141::aid-sim479>3.0.co;2-f 10797513

[26] Yu G Wang L Han Y He Q. clusterProfiler: an R package for comparing biological themes among gene clusters. *Omics.* (2012) 16:284–7. 10.1089/omi.2011.0118 22455463 PMC3339379

[27] Shen B Mei M Pu Y Zhang H Liu H Tang M Necrostatin-1 attenuates renal ischemia and reperfusion injury via meditation of HIF-1α/mir-26a/TRPC6/PARP1 signaling. *Mol Ther Nucleic Acids.* (2019) 17:701–13. 10.1016/j.omtn.2019.06.025 31422287 PMC6706591

[28] Liu H Wang L Weng X Chen H Du Y Diao C Inhibition of Brd4 alleviates renal ischemia/reperfusion injury-induced apoptosis and endoplasmic reticulum stress by blocking FoxO4-mediated oxidative stress. *Redox Biol.* (2019) 24:101195. 10.1016/j.redox.2019.101195 31004990 PMC6475721

[29] Szklarczyk D Gable AL Lyon D Junge A Wyder S Huerta-Cepas J STRING v11: protein-protein association networks with increased coverage, supporting functional discovery in genome-wide experimental datasets. *Nucleic Acids Res.* (2019) 47:D607–13. 10.1093/nar/gky1131 30476243 PMC6323986

[30] Mehta RL Cerdá J Burdmann EA Tonelli M García-García G Jha V International society of nephrology’s 0by25 initiative for acute kidney injury (zero preventable deaths by 2025): a human rights case for nephrology. *Lancet.* (2015) 385:2616–43. 10.1016/S0140-6736(15)60126-X 25777661

[31] Zeng X Mcmahon GM Brunelli SM Bates DW Waikar SS. Incidence, Outcomes, and Comparisons across Definitions of AKI in Hospitalized Individuals. *Clin J Am Soc Nephrol.* (2014) 9:12–20. 24178971 10.2215/CJN.02730313 PMC3878695

[32] Nangaku M Hirakawa Y Mimura I Inagi R Tanaka T. Epigenetic changes in the acute kidney injury-to-chronic kidney disease transition. *Nephron.* (2017) 137:256–9. 28595179 10.1159/000476078

[33] Wragg NM Burke L Wilson SL. A critical review of current progress in 3D kidney biomanufacturing: advances, challenges, and recommendations. *Renal Replace Ther.* (2019) 5:1–16.

[34] Wei J Haddad A Wu K Zhao H Kapur P Zhang Z A CpG-methylation-based assay to predict survival in clear cell renal cell carcinoma. *Nat Commun.* (2015) 6:8699. 10.1038/ncomms9699 26515236 PMC4846314

[35] El-Dahr S Hilliard S Saifudeen Z. Regulation of kidney development by the Mdm2/Mdm4-p53 axis. *J Mol Cell Biol.* (2017) 9:26–33. 10.1093/jmcb/mjx005 28096292 PMC5907835

[36] Jiang K Sun F Zhu J Luo G Ban Y Zhang P. miR-33a inhibits cell growth in renal cancer by downregulation of MDM4. *Mol Genet Genom Med.* (2019) 7:e833. 10.1002/mgg3.833 31250570 PMC6687894

[37] Li F Aljahdali IAM Zhang R Nastiuk KL Krolewski JJ Ling X. Kidney cancer biomarkers and targets for therapeutics: survivin (BIRC5), XIAP. *J Exp Clin Cancer Res.* (2021) 40:254. 10.1186/s13046-021-02026-1 34384473 PMC8359575

[38] Hilliard SA Li Y Dixon A El-Dahr SS. Mdm4 controls ureteric bud branching via regulation of p53 activity. *Mech Dev.* (2020) 163:103616. 10.1016/j.mod.2020.103616 32464196 PMC7487065

[39] Cui Z Xia Y Ye Y Jiang Z Wang Y Wu J RALY RNA binding protein-like reduced expression is associated with poor prognosis in clear cell renal cell carcinoma. *Asian Pac J Cancer Prev.* (2012) 13:3403–8. 10.7314/apjcp.2012.13.7.3403 22994768

[40] Everaert C Luypaert M Maag JLV Cheng QX Dinger ME Hellemans J Benchmarking of RNA-sequencing analysis workflows using whole-transcriptome RT-qPCR expression data. *Sci Rep.* (2017) 7:1559. 10.1038/s41598-017-01617-3 28484260 PMC5431503

[41] Nalewajska M Gurazda K Marchelek-Myśliwiec M Pawlik A Dziedziejko V. The role of endocan in selected kidney diseases. *Int J Mol Sci.* (2020) 21:6119. 10.3390/ijms21176119 32854332 PMC7504273

[42] Li S Wang L Wang C Wang Q Yang H Liang P Detection on dynamic changes of endothelial cell specific molecule-1 in acute rejection after renal transplantation. *Urology.* (2012) 80:738.e1–8. 10.1016/j.urology.2012.03.019 22608803

[43] Pulkkinen K Murugan S Vainio S. Wnt signaling in kidney development and disease. *Organogenesis.* (2008) 4:55–9. 19279716 10.4161/org.4.2.5849 PMC2634248

[44] Sebio A Kahn M Lenz HJ. The potential of targeting Wnt/beta-catenin in colon cancer. *Expert Opin Ther Targets.* (2014) 18:611–5. 24702624 10.1517/14728222.2014.906580

[45] Zhou D Li Y Lin L Zhou L Igarashi P Liu Y. Tubule-specific ablation of endogenous β-catenin aggravates acute kidney injury in mice. *Kidney Int.* (2012) 82:537–47. 10.1038/ki.2012.173 22622501 PMC3425732

