# Supplementary figures and images for: Machine learning algorithm-based identification and verification of characteristic genes in acute kidney injury
Source: Front Med (Lausanne). 2022 Oct 13;9:1016459. doi: 10.3389/fmed.2022.1016459 (PMC9606399; doi:10.3389/fmed.2022.1016459)

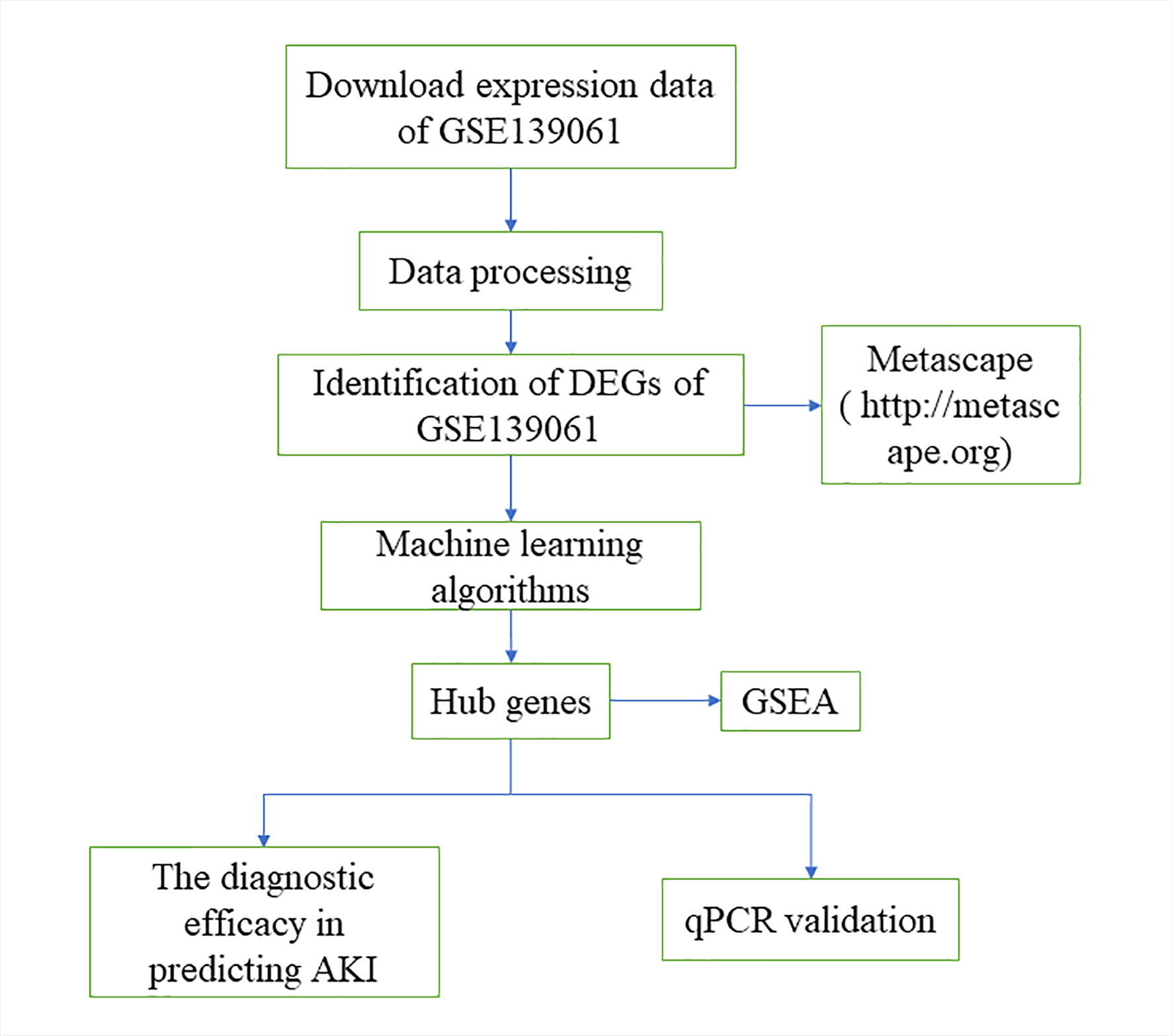

Supplement: Supplementary file 5 [file Image_1.TIF]

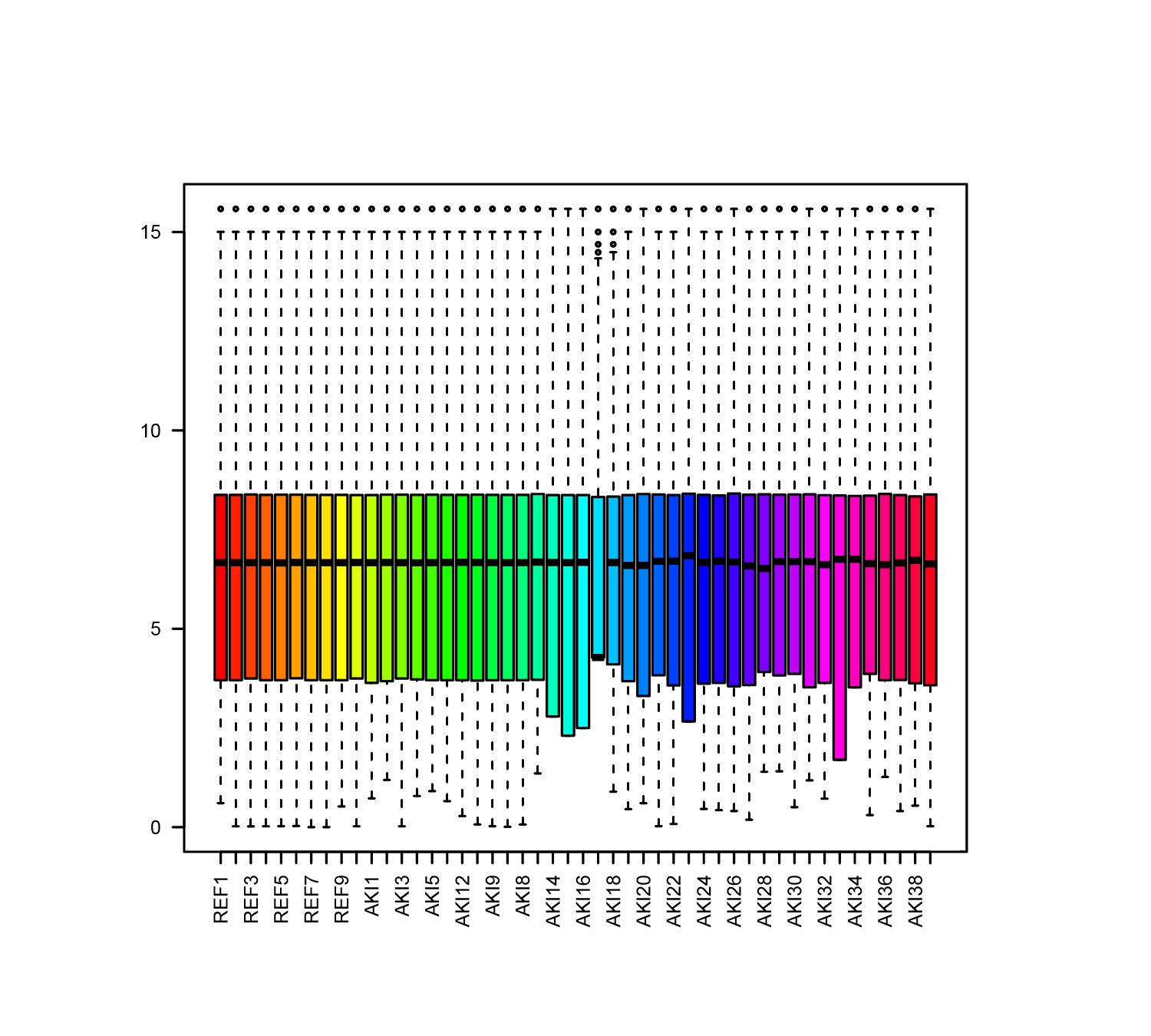

Supplement: Supplementary file 6 [file Image_2.TIF]

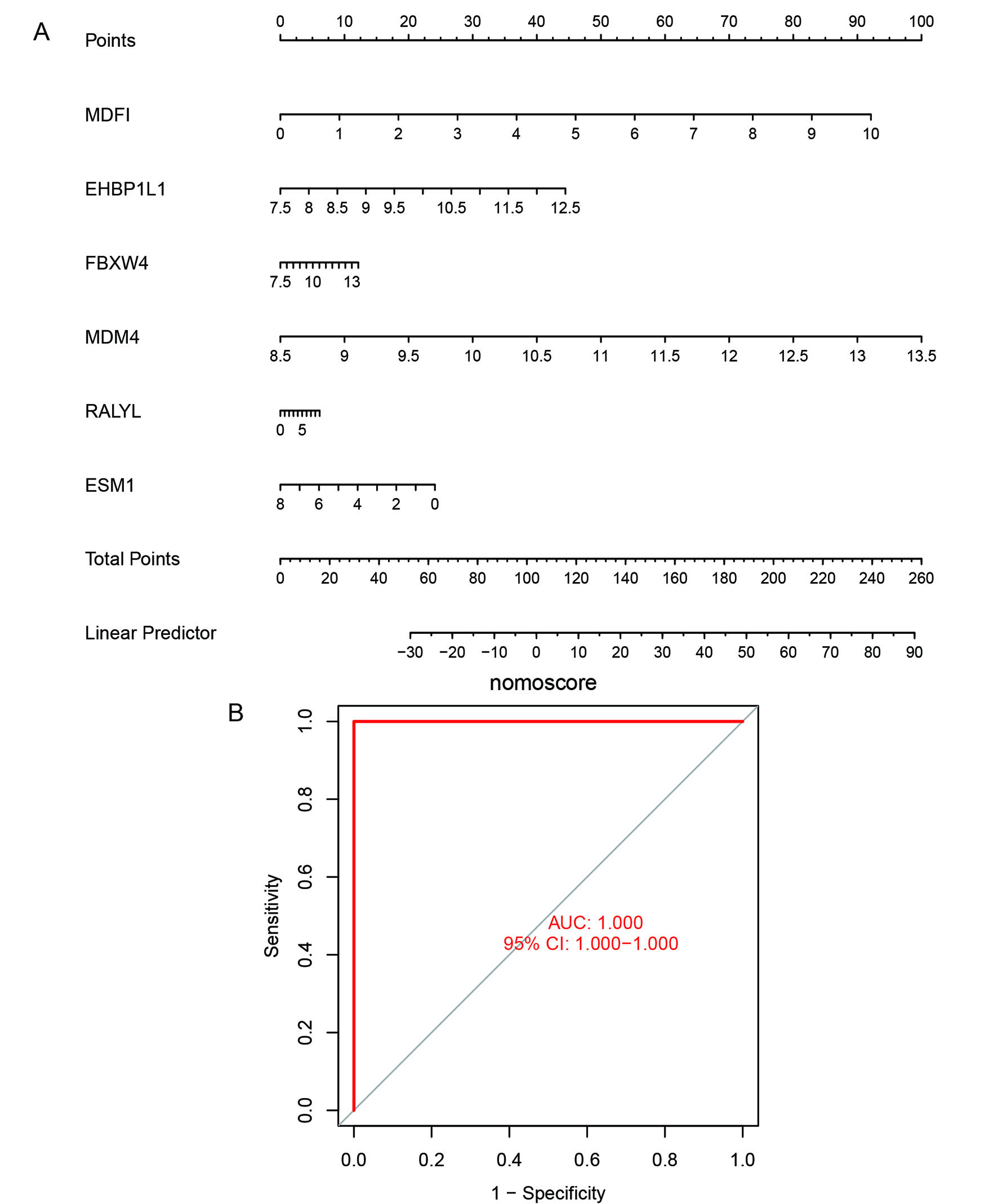

Supplement: Supplementary file 7 [file Image_3.TIF]

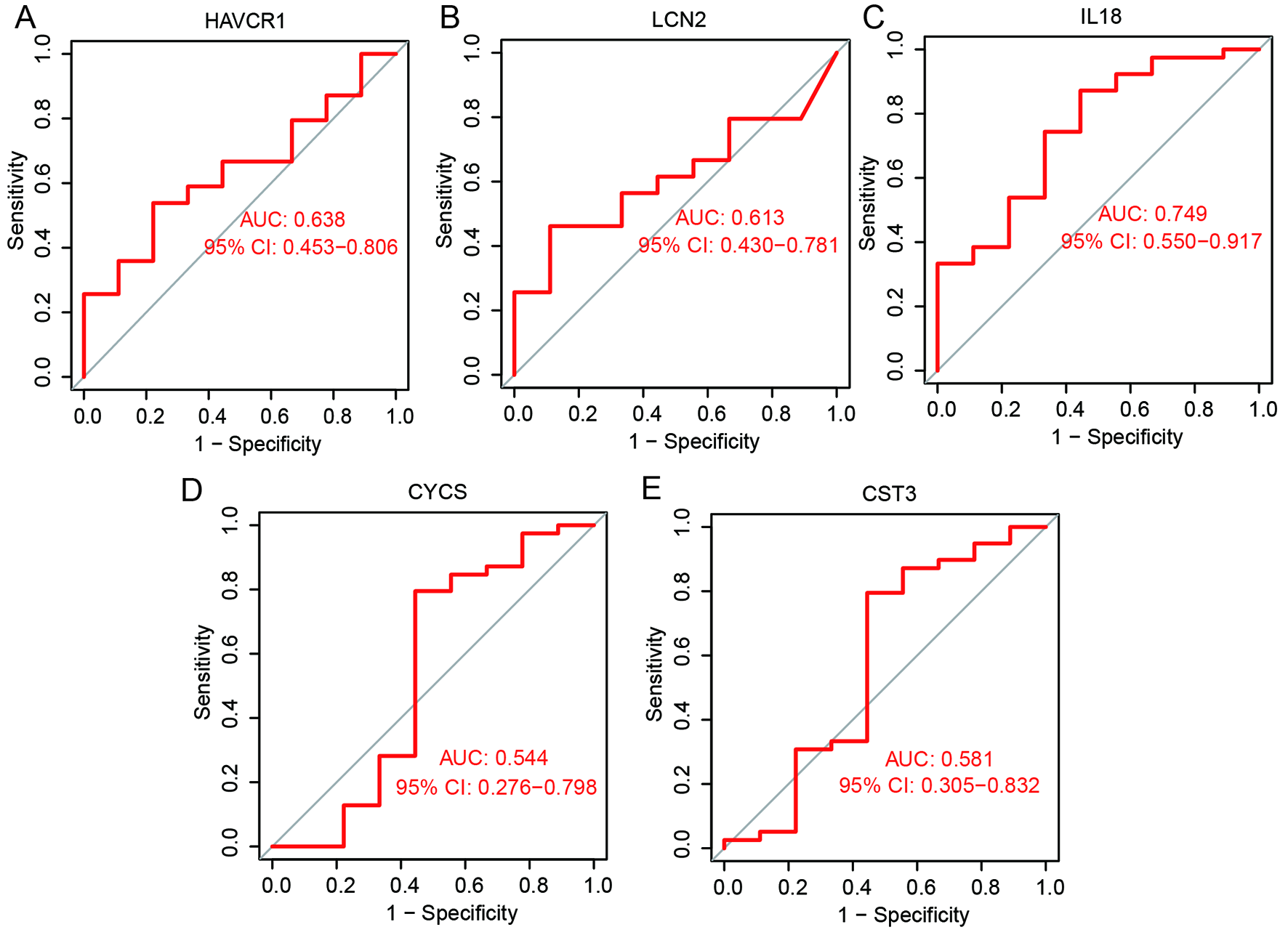

Supplement: Supplementary file 8 [file Image_4.TIF]
